# Mechanical Properties and Corrosion Resistance of Thin Ceria and Phosphate Mono- and Multilayers Deposited on Technically Pure Al 1050

**DOI:** 10.3390/ma18020424

**Published:** 2025-01-17

**Authors:** Sabina Cherneva, Reni Andreeva, Dimitar Stoychev

**Affiliations:** 1 Institute of Mechanics, Bulgarian Academy of Sciences, Acad. G. Bonchev St., Bl.4, 1113 Sofia, Bulgaria; sabina_cherneva@yahoo.com; 2 Institute of Physical Chemistry, Bulgarian Academy of Sciences, Acad. G. Bonchev St., Bl.11, 1113 Sofia, Bulgaria; randreeva@ipc.bas.bg

**Keywords:** aluminum, anodization, ceria/phosphate conversion coatings, corrosion, mechanical properties, nanoindentation

## Abstract

Calcium phosphates are often used for biomedical applications. Hydroxyapatite, for example, has a wide range of applications because it mimics the mineral component of natural bone. Widespread interest in the catalytic properties of ceria is due to its use in automotive catalytic converters. Effect of electroless deposited on (non-anodized and anodized) Al 1050 with monolayer Ce_2_O_3_ + CeO_2_, consecutive deposited bilayer Ce_2_O_3_ + CeO_2_/Ca_5_(PO_4_)_3_OH or consecutive deposited bilayer Ce_2_O_3_ + CeO_2_/(AlPO_4_ + AlOOH + CePO_4_) systems on the indentation modulus (E_IT_) and hardness (H_IT_), as well as their corrosion-protective ability were investigated. For structural, chemical, electrochemical, and mechanical characterization of the investigated systems, the following methods were used: scanning electron microscopy (SEM), energy dispersive X-ray analysis (EDXS), X-ray photoelectron spectroscopy (XPS), polarization resistance (R_p_), corrosion rate (CR) analysis, and nanoindentation. It was found that the H_IT_ and E_IT_ of the coatings deposited on an anodized aluminum substrate were much higher than those deposited on a non-anodized aluminum substrate. It established a specific influence of the morphology and chemical composition of formed conversion layers on H_IT_ and E_IT_ and improved the corrosion-protective effect of these layers. The obtained results are valuable since there is no data on the mechanical properties of such coatings in the literature to date.

## 1. Introduction

Deposition of mono- and multilayer functional coatings on different metal substrates is a commonly accepted practice. In particular, corrosion-protective metal and/or oxide layers applied on corrosion-prone metal and alloy substrates have wide applications. Each protective layer has its specific function. For example, the influence of widely applied ceria layers deposited on different Al alloys is well documented [1,2,3,4,5,6,7,8,9,10,11,12,13,14]. The deposition of cerium oxide conversion coatings (COCCs) on Al is a potential alternative to [15,16,17] highly effective yet highly toxic and carcinogenic Cr6+-containing conversion layers [18]. It is necessary to note that the ceria conversion layers, upon drying (due to the removal of crystal water in the ceria layers) [19,20], develop cracks and pores, which to a certain extent reduce their corrosion-protective ability. To mitigate this effect, some studies and successful solutions have been carried out based on the modification of the COCC with various direct [21] or post-treatment operations using Na_3_PO_4_, NH_4_H_2_PO_4_, NaH_2_PO_4_, etc. solutions [22,23,24,25].

Recently, the highly effective application of hydroxyapatite/Ca_5_(PO_4_)_3_OH (hereafter referred to as CaPhL) as a chemically deposited corrosion-protective coating on aluminum (deposited on a steel substrate by the wire arc sprayed aluminum coatings process) was investigated and proven. In this case, direct immersion phosphating of aluminum (in mixed NaH_2_PO_4_ and Ca(NO_3_)_2_ solution) was applied, which was carried out without prior formation of COCC on it [26,27,28,29]. In subsequent studies aimed at reducing energy consumption and increasing the environmental friendliness of technological processes, the positive protective effect of CaPhL was also confirmed in the deposition of much thinner conversion layers on (non-anodized and anodized) Al 1050 aluminum substrates. Moreover, the deposition of CaPhL was combined with the prior formation of a protective COCC layer on this substrate [30,31]. In parallel to these studies, results comparing the corrosion-protective behavior of phosphate coatings formed on Al 1050 during its chemical surface treatment in NH_4_H_2_PO_4_ solution [24] (hereafter referred to as NH_4_PhL) were obtained [24].

The studies noted above characterize some of the possibilities for the successful replacement of environmentally unsuitable and toxic corrosion-protective coatings on aluminum through the application and combination of ceria and phosphate conversion coatings. The assessments of these capabilities are based on the analysis of the changes occurring in their physicochemical properties, established in the publications cited above. These assessments are not able to fully characterize their protective capabilities if some of their physicomechanical properties are not quantitatively investigated. This assumption is related to the above-mentioned ceria and phosphate layers modifying the surface of Al 1050, which are characterized by specific structural features and defects. In the context of these considerations, the search and establishment of possible interrelations between the physicochemical and physicomechanical properties of the studied systems is necessary. They can be established, clarified, and commented on in cases where the occurring changes in the morphology, structure, chemical, and phase composition as well as some of the physicomechanical properties of the studied systems are analyzed and compared explicitly in qualitative and quantitative aspects. An initiating example is the result presented in [32,33], in which results were obtained and commented on, convincingly illustrating correlations between microstructure, hardness, and corrosion. There is a lack of data on the mechanical properties of ceria/phosphate coatings in the literature. We found such data only for hydroxyapatite [34]. The novelty of our current study lies in addressing these gaps in the literature.

The aim of the present work was to characterize, through nanoindentation experiments, two of the main mechanical properties of the mono- and bilayer conversion coatings discussed above—indentation modulus (E_IT_) and indentation hardness (H_IT_). The results obtained from these measurements were compared with the data from the physicochemical and corrosion studies carried out on the same samples.

## 2. Materials and Methods

The cerium oxide(s) (Ce_2_O_3_ and CeO_2_) conversion coatings (CОСC), obtained by chemical immersion treatment, were deposited on substrates of “technically pure” Al 1050 (containing 0.40% Fe, 0.25% Si, 0.05% Mn, 0.05% Cu, 0.07% Zn, 0.05% Mg). This material was selected as a model object by us as it finds a wide range of applications as a construction material. The studied samples, measuring 2.5 × 2.5 × 0.1 cm, were cut from rolled Al 1050 sheets. Their pre-treatment, as described in [7], involved consecutive degreasing in an organic solvent and etching in 1.5 M NaOH (these samples/substrates are hereafter abbreviated as “S1”; see also Table 1).

In the case where S1 is additionally anodized in a solution of sulfuric acid (15% H_2_SO_4_; 15 °C; 1.5 A.dm^2^; 48 min; the thickness of Al_2_O_3_ ≈ 18 µm), the abbreviation of sample is marked by “`”, i.e., as S1`.

The formation of COCC on the Al substrate (S1) was realized in 0.5 M CeCl_3_ × 7H_2_O + 1 × 10^−5^ M CuCl_2_ × 2H_2_O (at 25 °C for an immersion time of 2 h). No H_2_O_2_ or other types of oxidizing agents were added [6,7]. The abbreviation of these samples in the following text is “S2”. In the cases when S1 is additionally anodized, the abbreviation is “S2`”. We used XPS depth profiling of deposited COCC [7] to measure its thickness. We found that it was 110 nm.

After the formation of COCC, the specimens were rinsed in distilled water, dried, and post-treated in mixed 0.5 M NaH_2_PO_4_ + 0.1 M Ca(NO_3_)_2_ solution, according to the procedure described in [30], before exposure and investigation in model corrosion medium of 0.1 M NaCl (CM) at 25 °C in the time interval 0.25 till 168 h. The phosphate layers were 1 µm thick (the thickness was determined by SEM of the samples’ cross-sections). The abbreviation of these samples in the following text is “S3”. In the cases when S1 substrate is additionally anodized, the abbreviation is “S3`”. A similar procedure was applied in the case of Al substrate(s) (non-anodized or anodized) after the formation of COCC, which were post-treated in a solution of 0.22 M NH_4_H_2_PO_4_ (Alfa Aesar, Haverhill, MA, USA), as applied and studied in detail in [24]. The abbreviations of these samples in the further text are “S4” and “S4`”, respectively (Table 1).

We investigated the surface morphology, structure, and chemical composition of the samples using electron microscopy (JEOL JSM 6390, Tokyo, Japan) with secondary electron imaging (SEM) and characteristic energy-dispersive X-ray spectroscopy (EDXS) (AZtecOne, Oxford Instruments, Abingdon, UK). The quantitative EDXS data were obtained with ZAF correction and using external standards [7,24,25]. We performed X-ray photoelectron spectroscopy (XPS) using an AXIS Supra electron spectrometer (Kratos Analytical Ltd., Manchester, UK) with achromatic Al-Kα radiation at a photon energy of 1486.6 eV. The investigated area was 0.3 × 0.75 mm^2^. The energy calibration was performed by fixing the C1s line of adsorbed adventitious hydrocarbons to 284.6 eV. The binding energies (BE) were measured with an accuracy of ±0.1 eV. The changes in composition and chemical surroundings of the chemical elements were determined by monitoring the areas and binding energies of the photoelectron peaks of the appropriate elements, that is, C1s, O1s, Ce3d, etc. The chemical composition and the state of the elements of the investigated coatings and substrates are shown in Section 3.

We investigated the corrosion rate (R_p_, Ω cm^2^) and polarization resistance (CR, μm/year) of the samples (shown in Table 5) using Gamry Interface 1000 (Software and Frequency Response Analyzer EIS300, Warminster, PA, USA, 2021). The initial delay was 15 min, the scan range was 15 mV, and the temperature was 25 °C ± 0.5 °C. The Stern–Geary equation was used for the calculation of the Rp of the coatings [35], because a higher Rp value (in Ω cm^2^) corresponds to higher corrosion resistance and lower corrosion current (i_cor_), i.e., R_p_~1/i_cor_. To check the reproducibility, the Rp and CR measurements were repeated at least three times. The mechanical properties (H_IT_ and E_IT_) of electroless deposited (on non-anodized and anodized technically pure Al 1050 substrate(s)) thin mono- and multilayer (based on mixed compounds: Ce_2_O_3_ + Ce_2_O_4_—b and b` [6,7]; Ca_5_(PO_4_)_3_(OH) + CePO_4_ + AlPO_4_ + Al(OH)_3_—c and c`; AlPO_4_ and AlOOH, CePO_4_, as well as PO^3−^, P_2_O_5_, and P_4_O_10_ compounds with Al and Се—d and d` [23,24,25,30,31]) coatings shown in Section 3.3 were investigated using nanoindentation experiments under the ISO 14577 standard [36]. The equipment used for the nanoindentation experiments was a Nanoindenter G200 (KLA Corporation, Milpitas, CA, USA) with a Berkovich diamond tip with a 20 nm tip rounding. We used the so-called “XP/G-Series Basic Hardness, Modulus at a Depth” indentation method, which has a depth control. The parameters used for nanoindentation testing were the following: 60 nm depth limit, 10 s peak hold time, 90% to unload, 10 nm/s surface approach velocity, 5000 nm surface approach distance, 40% surface approach sensitivity, 50% unload percentage in stiffness calculation, and 0.3 Poisson’s ratio. Twenty-five indentations of all investigated samples were made.

## 3. Results and Discussion

### 3.1. SEM, EDS, and XPS Data

**Figure 1:**
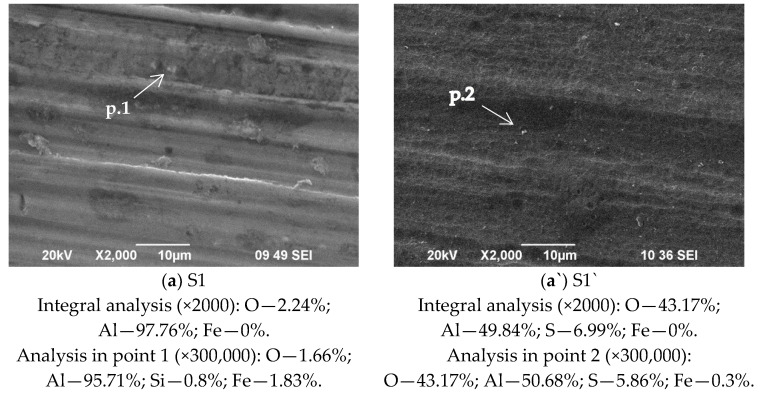

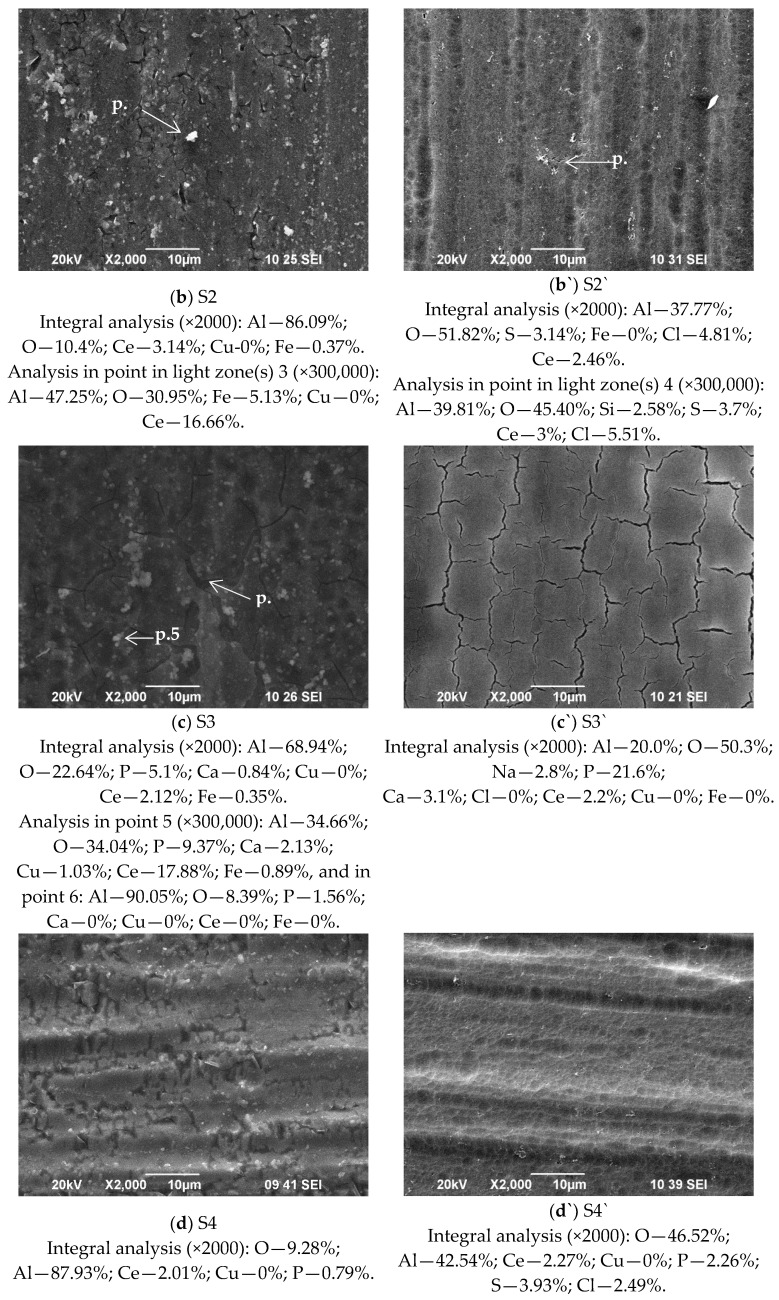
SEM images and EDS analysis of the studied samples after (**a**) pre-treatment of Al 1050 in NaOH (Al_(NaOH)_); (**b**) immersion treatment of Al_(NaOH)_ in 0.5 M CeCl_3_ × 7H_2_O + 1 × 10^−5^ M CuCl_2_ × 2H_2_O; (**c**) consecutively immersion treatment of Al_(NaOH)_ in 0.5 M CeCl_3_ × 7H_2_O + 1 × 10^−5^ M CuCl_2_ × 2H_2_O and 3 × 5 min in mixed 0.5 M NaH_2_PO_4_ + 0.1 M Ca(NO_3_)_2_ solution and 72 h exposure in humidity atmosphere; (**d**) consecutively immersion treatment of Al_(NaOH)_ in 0.5 M CeCl_3_ × 7H_2_O + 1 × 10^−5^ M CuCl_2_ × 2H_2_O and 5 min in NH_4_H_2_PO_4_ solution; (**a`**) pre-treatment in NaOH and anodized Al 1050 (Al_anod_); (**b`**) immersion treatment of Al_anod_ in 0.5 M CeCl_3_ × 7H_2_O + 1 × 10^−5^ M CuCl_2_ × 2H_2_O; (**c`**) consecutively immersion treatment of Al_anod_ in 0.5 M CeCl_3_ × 7H_2_O + 1 × 10^−5^ M CuCl_2_ × 2H_2_O and 3 × 5 min in mixed 0.5 M NaH_2_PO_4_ + 0.1 M Ca(NO_3_)_2_ solution and 72 h exposure in humidity atmosphere; (**d`**) consecutively immersion treatment of Al_anod._ in 0.5 M CeCl_3_ × 7H_2_O + 1 × 10^−5^ M CuCl_2_ × 2H_2_O and 5 min in NH_4_H_2_PO_4_ solution.

SEM images of the surfaces of investigated layers, deposited on Al 1050 substrate after its pre-treatment in 1.5 M NaOH—(a)—sample S1 and after anodization of S1 in 15% H_2_SO_4_—(a`)—sample S1` (see Table 1) are shown in Figure 1. Their immersion treatment in a solution of 0.5 M CeCl_3_ × 7H_2_O + 1 × 10^−5^ M CuCl_2_ × 2H_2_O are marked in Figure 1 as (b) S2 and (b`) S2`, respectively. The samples S2 and S2` followed by post-treatment sealing in mixed 0.5 M NaH_2_PO_4_ + 0.1 M Ca (NO_3_)_2_ are marked as (c) and (c`)—S3 and S3`, respectively. The samples S2 and S2` followed by sealing post-treatment in 0.22 M NH_4_H_2_PO_4_ solutions are marked in Figure 1 as (d) and (d`)—S4 and S4`, respectively.

From the presented results, it appears that the alkaline etching of Al 1050 (Figure 1a) and its anodization (Figure 1a`) to the greatest extent are built of spherical agglomerates. They are evenly distributed over the entire surface, replicating the profile of Al substrates. Therefore, the roughness and hardness determined by the mechanical processing of the rolled Al 1050 sheet/substrate affect the formation of chemically deposited COCC in a specific way [7]. The results presented in Figure 1a,a`,b` show that the formed COCC on the anodized Al (the system S2/b`) essentially reproduces the anodized Al substrate’s (S1/a`) topology—a conclusion that was also established and elucidated in [37,38]. The structural changes of sealing post-treated samples S2 and S2` in mixed 0.5 M NaH_2_PO_4_ + 0.1 M Ca(NO_3_)_2_ solution lead to the formation of a calcium phosphate layer (CaPhL) on S3 and S3` [30]. Moreover, its morphology and structure differ significantly from that of the COCC layers deposited on S2 and S2` (Figure 1b,b`). In S3, the agglomerates forming the phosphate layer are larger and more strongly striated by cracks (Figure 1c) compared to the ceria layers formed in S2 (Figure 1b). At the same time, the deposited CaPhL layers on sample S2`—the S3` system—are built up of a regular network of cracks, the width of which is several times smaller (Figure 1c`) compared to the S3 system. Specific are the changes of the surface morphology and structure of (coated by COCC) samples S2 and S2` after their sealing post-treated in 0.22 M NH_4_H_2_PO_4_ solution (forming NH_4_PhL layer). The results obtained in this case are shown in Figure 1d,d`. From them, it can be seen that the deposited NH_4_PhLs layers reproduce the morphology of the non-anodized and anodized aluminum substrates S1 and S1` (Figure 1a,a`). The NH_4_PhL deposited on S2 (Figure 1b) contains numerous pores and cracks (Figure 1d), whereas, on the S2, i.e., on the anodized substrates, similar defects are not observed (see Figure 1a`,b`,d`).

The chemical composition in the bulk of the studied systems determined by EDS analysis (in weight %) is shown in Table 2 (and under the photographs in Figure 1). In parallel with these studies, XPS analyses (Table 3) were performed on the same samples, illustrating the surface concentration (in atomic %) and the chemical state of the elements constituting the formed conversion coatings. By duplicating and comparing the results of the two analysis methods, we aimed to establish possible differences in the chemical composition and chemical state of the elements along the depth of the conversion layers: on the surface (the zone in which the corrosion processes mainly develop) and in depth (the zone in which the chemical and phase compositions determine their physicomechanical properties).

The comparison of the changes in the concentrations of the elements Ce, Ca, and P characteristic of the formed conversion layers, registered in the EDS analyses (Table 2), shows that the concentration of Ce changes insignificantly in all the studied samples, varying in the range of 2.0–3.1 wt. %. About four times greater (on the anodized substrates) is the change in the Ca content in samples S3 and S3` (CS3:CS3` = 0.8:3.1%), while the change in the concentration of P is even more pronounced in samples S3 and S3` (CS3:CS3` = 5.1:21.6%) and to a lesser extent in samples S4 and S4` (CS4:CS4` = 0.8:2.3%). The registered presence of Fe, S, and Cl is due to the manifestation of traces of Fe as a component of Al 1050; residual traces of S, after washing the anodized Al 1050 in H_2_SO_4_, are due to the difficult washing of H_2_SO_4_ from the pores of the anodized samples; residual traces of Cl^−^ are a component of the ССОС deposited on the studied samples.

The results of XPS analyses (Table 3) characterize the concentration distribution of the characteristic elements of the conversion cerium oxide and phosphate coatings in a somewhat different manner. The analysis of the obtained data shows that the concentration of Ce on the surface of S2 is 15.8 at.%, while on the anodized Al—S2`—it is more than fifteen times lower (0.9 at.%). In samples S3 and S3`, the concentration of Ce decreases to 0.5 and 0.4 at.%, respectively, while the concentration of Ca in both samples (unanodized and anodized) is 1.7 at.%. The P concentrations are 11.6 (on unanodized substrate) and 17.9 at.% (on anodized substrate), respectively. XPS analysis of these samples also registered the presence of the elements Na (0.6 at.% for S3 and 0.2 at.% for S3`) and N (1.0 at.% for S3 and 2.1 at.% for S3`). The registration of these two elements also indicates the presence of additional Na- and N-containing phases on the surface of the conversion layers of the systems S3 and S3` (Table 4). In samples S4 and S4`, subjected to sealing post-treatment only in 0.22 M NH_4_H_2_PO_4_, the concentration of cerium in the cerium-containing phases is very close—1.4 at.% in S4 and 1.3 at.% in S4`. The concentration of P is 14.1 at.% in S4 and 6.5 at.% in S4`. In sample S4`, the presence of 4.5 at.% N is also registered in phases in which N is present (see Table 4).

Based on the results obtained from XPS analyses of the chemical composition and chemical state of the elements found in the studied samples, the most probable phases that were formed during the directed preparation of the modeled corrosion protection systems on Al 1050 were defined. The chemical formulas of these phases are presented in Table 4. According to these data, the physicomechanical (H_IT_ and E_IT_) and physicochemical (Rp and CR) properties, and consequently the behavior, of the studied samples depend on the presence and concentrations of Al_2_O_3_, Се_2_O_3_, and СеО_2_ for S2 и S2`; Al_2_O_3_, AlPO_4_, AlOOH, Се_2_O;_3_, CePO_4_, NaH_2_PO_4_, Ca(PO_4_)_3_(OH), and Ca(NO_3_)_2_ for S3 and S3`; AlPO_4_, CePO_4_, PO_3_^−^, P_2_O_5_(P_4_O_10_), and NaH_2_PO_4_ for S4 and S4`.

The changes in concentration of the ceria, calcium, and phosphorous established in the bulk (EDS) of studied systems are shown in Table 2. Based on the obtained results, there is a set in lowering order for each of the investigated samples:

Ce: S2 > S2` > S4` > S3 = S3` > S4;
Ca: S3` > S3;
P: S3` > S3 > S4` > S4.

A similar characterization can describe the concentration changes of the same elements on the surface (XPS) of the chemically obtained systems included in Table 3.

Ce: S2 > S4 > S4` > S2` > S3` > S3`;
Ca: S3 = S3`;
P: S3` > S4 > S3;
Na: S3 > S3`;
N: S4` > S3` > S3.

### 3.2. Polarization Resistance and Corrosion Rate of the Studied Systems

**Figure 2:**
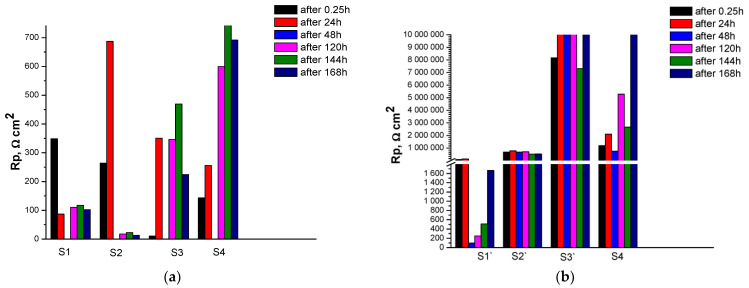
Change in Rp vs. time of exposure (for 0.25 h, 24 h, 48 h, 120 h, 144 h, and 168 h) in the CM of the samples: (**a**) S1, S2, S3, S4 (obtained on non-anodized Al 1050 substrates); (**b**) S1`, S2`, S3`, S4` (obtained on anodized Al 1050 substrates).

The results for the corrosion resistance R_p_ and the corrosion rate CR for the studied systems are presented in Figure 2 and Table 5. Figure 2a shows the results for the change in R_p_ for samples S1–S4 (formed on non-anodized Al 1050) with a standard increase in the exposure time of the samples (up to 168 h) in CM. It can be seen that the highest value for R_p_ at the initial moment of exposure (till 15 min) is characterized by sample S1 (R_p_ ≈ 350 Ωcm^2^), while the most resistant and reaching the highest value after 168 h of exposure in CM is sample S4 (Rp ≈ 690 Ωcm^2^). For samples S1`–S4` (formed on anodized Al 1050 substrates), both in terms of absolute value of R_p_ and in terms of resistance, upon prolonged exposure to CM, the results differ by orders of magnitude (Figure 2b). It can be seen that in the initial moments of exposure (till 15 min–24 h), R_p_ for S1` is characterized by values of the order of ≈8.5 × 10^5^ Ω cm^2^), after which it decreases sharply, and after only 168 h of exposure in CM, it reaches ≈ 1.8 × 10^3^ Ω cm^2^. Sample S3` (≈10 × 10^6^ Ω cm^2^) has the highest and relatively weak (up to the 168th hour of exposure in the CM) changing values for R_p_. Compared to S3`, sample S4`, at the initial moment of exposure in CM, is characterized by a value of the order of 1.4 × 10^6^ Ω cm^2^, which gradually increases and only after the 168th hour of exposure reaches ≈10 × 10^6^ Ω cm^2^. R_p_ of sample S2` practically does not change throughout the entire time interval of exposure in CM, characterized by values R_p_ ≈ 0.6 × 10^6^ Ω cm^2^.

**Table 5:** Changes in the Rp and CR values depending on the type of Al substrate(s) (non-anodized or anodized) and the time of exposure in the corrosion medium (as deposited and after 168 h in CM).

Sample		Rp, Ω.cm^2^	CR, µm/year
S1	As deposited	3.5 × 10^2^	0.8
	After 168 h in CM	1.0 × 10^2^	2.8
S1`	As deposited After 168 h in CM	0.9 × 10^5^ 1.7 × 10^3^	1.5 × 10^−5^ 2.0 × 10^−1^
S2	As deposited	2.6 × 10^2^	1.1
	After 168 h in CM	1.4 × 10^1^	21
S2`	As deposited After 168 h in CM	6.8 × 10^5^ 5.3 × 10^5^	4.2 × 10^−4^ 5.4 × 10^−4^
S3	As deposited After 168 h in CM	1.1 × 10^1^ 2.2 × 10^2^	25 1.3
S3`	As deposited After 168 h in CM	8.2 × 10^6^ 2.3 × 10^7^	3.5 × 10^−5^ 1.2 × 10^−5^
S4	As deposited After 168 h in CM	1.4 × 10^2^ 6.9 × 10^2^	2.0 0.4
S4`	As deposited After 168 h in CM	1.2 × 10^6^ 6.9 × 10^7^	2.4 × 10^−4^ 4.1 × 10^−6^

The changes in the corrosion rate (CR) of the studied samples and the values for Rp (compared in numerical terms) as a function of the exposure time in CM (systematized in Table 5) show the following:

‑ For the non-anodized Al 1050 substrate (sample S1), Rp (after 15 min and 168 h of exposure in CM) decreases about three and a half times (3.5 × 102 vs. 1.0 × 102), which leads to a more than threefold increase in CR (0.8 vs. 2.8 µm/y). The sealing treatment in ceria-containing solution (leading to the formation of COCC on S1–sample S2) decreases Rp about more of an order (2.6 × 102 vs. 1.4 × 101) which leads to the fact of about twenty times (1.1 vs. 21 µm/year) increase of CR.
‑ For the sample S3, Rp decreases strongly (1.1 × 101 vs. 2.2 × 102), and CR decreases about twenty times (25 vs. 1.3 µm/y).
‑ For the sample S4, Rp increases about five times (1.4 × 102 vs. 6.9 × 102), and CR decreases five times (2.0 vs. 0.4 µm/y).

Similar but more pronounced trends are observed for the samples S1`, S2`, S3`, and S4`, whose values are much higher (see Table 5).

The variation of R_p_ and CR (by up to 7 orders of magnitude—Table 5) depending on whether the studied protective conversion systems are implemented on a non-anodized or anodized substrate is impressive. An interesting example in this regard is samples S2 and S2`. While CR for as-deposited samples S2 increases ~ 20 times after 168 h of exposure in CM, for sample S2`, this ratio in the corrosion rate is practically the same, although it is 4 orders of magnitude lower compared to the S2 system (effect more probably connected with the formation of non-soluble corrosion products on the sample’s surface). These results suggest that the cause of this effect is the availability of deep structural defects introduced to the protective COCC on sample S2 (Figure 1b and the absence of such on sample S2` (Figure 1b`). A significant contribution to this effect is also made by the Al_2_O_3_ layer formed during the anodization of Al 1050. This assumption is supported by the results (Figure 1c,c`,d,d`, obtained with additional post-sealing treatments, realized at S3 and S3`, and S4 and S4`, respectively, which blocked pores and cracks in COCC conversion layers [30,31]. Confirming this assumption are the results presented in Figure 2. Moreover, they provide grounds for the conclusion that for the non-anodized substrates (samples S1, S2, S3, and S4) the secondary sealing treatment of COCC by NH_4_H_2_PO_4_ solution (forming NH_4_PhL layer) (see sample S3 on Figure 2a) is more effective while for the anodized Al 1050 substrates sealed by COCC (S1`, S2`, S3`, and S4`) more effective is secondary sealing treatment in mixed NaH_2_PO_4_ + Ca(NO_3_)_2_ solution (forming CaPhL layer) (see sample S3` on Figure 2b).

### 3.3. Nanoindentation Investigations

**Figure 3:**
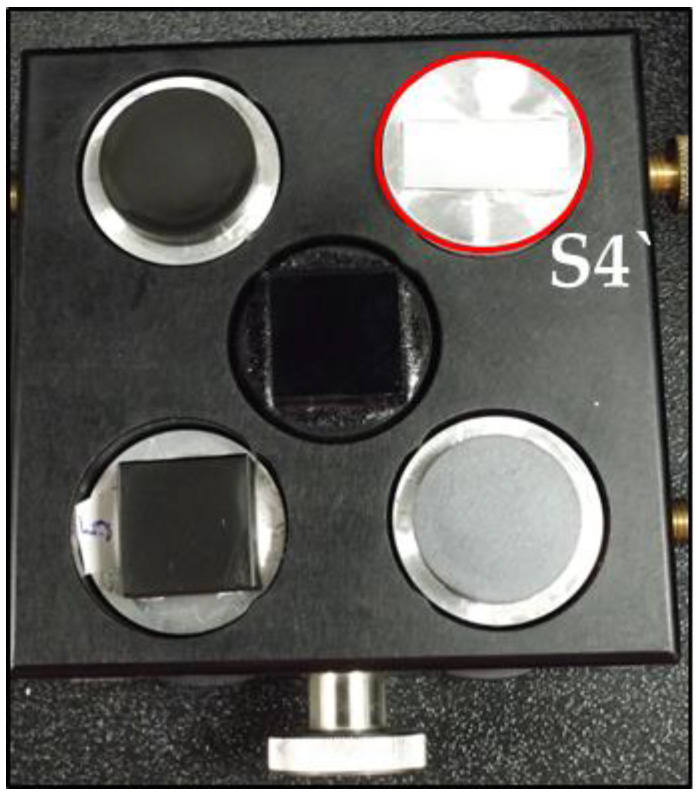
Tested samples, prepared for nanoindentation experiment.

**Figure 4:**
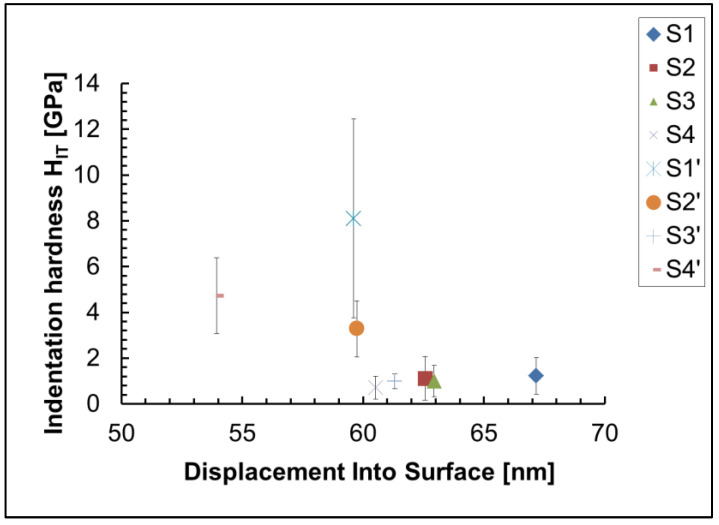
Indentation hardness of investigated films and substrates.

**Figure 5:**
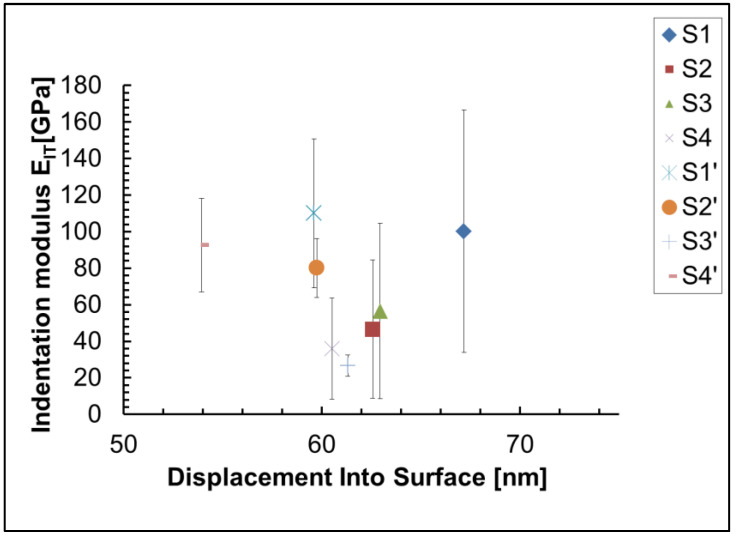
Indentation modulus of investigated films and substrates.

The sample S4`, prepared for nanoindentation experiments, is shown in Figure 3. We used the method of Oliver and Pharr [39] to calculate the indentation hardness H_IT_ and indentation modulus E_IT_ of investigated coatings. The results can be seen in Figure 4 and Figure 5. Our investigations showed that the indentation hardness and modulus of the coatings deposited on an anodized aluminum substrate were much higher than those deposited on a non-anodized aluminum substrate. The possible reason is the formed on the substrate surface hard Al_2_O_3_ film.

The mean value from 25 measurements of the indentation hardness of the non-anodized Al 1050 substrate is 1.234 ± 0.804 GPa, and its indentation modulus is 100.196 ± 66.359 GPa. The highest indentation hardness (8.104 ± 4.348 GPa) and modulus (110.02 ± 40.673 GPa) of all samples is found in sample S1` (the anodized substrate), due to the influence of the hard Al_2_O_3_ layer formed during the anodization of Al 1050 and the lack of soft ceria (mix of Ce_2_O_3_ and CeO_2_) layer in the composition, which is present in all investigated coatings. As the nanoindentation experiments have been made at a very small depth of indentation (only 60 nm), we do not expect to influence the substrate except for sample S2, where the thickness of the coating is 110 nm only. The influence of the hard anodized substrate is one of the reasons why sample S2` has a higher hardness (3.28 ± 1.22 GPa) than samples S1 (1.23 ± 0.80 GPa), S2 (1.11 ± 0.95 GPa), S3 (1.00 ± 0.691 GPa), S4 (0.71 ± 0.50 GPa), and S3` (0.99 ± 0.32 GPa). Since the concentration of soft ceria on the surface of S2 is 15.8 at.% (Table 3), while on the anodized Al—S2`—it is more than fifteen times lower (0.9 at.%), this is another reason for the higher hardness of sample S2` compared to S2, and a third factor that has an influence is the presence of pores and cracks in sample S2, which are not present in sample S2`. In samples S4 and S4`, subjected to sealing post-treatment only in 0.22 M NH_4_H_2_PO_4_, the concentration of cerium in the cerium-containing phases is 1.4 at.% in S4 and 1.3 at.% in S4` (Table 3). This is another reason for the greater hardness of sample S4` (4.72 ± 1.65 GPa) compared to S4 (Table 3). The presence of cracks, pores, and defects, as well as the phase composition, also influences the mechanical properties. S3` contains numerous pores and cracks, while in sample S4`, similar defects are not observed. This is probably one of the reasons why sample S4` is harder than S3` (Figure 1). Another possible reason why sample S4` is harder than S3` and S2` is the influence of the PO^3−^ and P_4_O_10_ phases, present only in sample S4`. Also, due to the three times lower concentration of soft aluminum in its composition, S4` is harder than S2`. It can be seen from the SEM images and EDS analysis of studied samples (Figure 1) that the chemical composition and content in different points (Figure 1, analysis “in points” 1, 2, 3, 4, 5, and 6 at high magnification—300,000×) on the sample’s surface is different, which means inhomogeneity of the sample surface, which is the main reason for the large confidence intervals of the experimental results of nanoindentation, which are visible on Figure 4 and Figure 5. Another reason is the high roughness of the sample surfaces.

We found literature data for nanoindentation of hydroxyapatite or calcium phosphate films primarily for biomedical applications, deposited mostly on titanium, silicon wafers, or steel substrates [40,41,42,43,44] using other deposition techniques (micro-arc oxidation, pulsed laser deposition, microplasma spraying, radio-frequency (RF)-sputtered). The different deposition techniques and substrates lead to different structures of the obtained films, resulting in different mechanical properties. In addition to the scarcity of literature data about the mechanical properties of such films, this highlights the importance of the present study. Our results on the hydroxyapatite films are in good agreement with the results obtained by Dey and coauthors [43], and the results for the Al 1050 alloy are similar to those obtained by El-Danaf, Qiao, Mohamed, and Yang [45,46,47,48].

## 4. Conclusions

In the present work, we investigated the mechanical properties and corrosion resistance of thin ceria and phosphate mono- and multilayers, deposited on technically pure Al 1050. SEM, EDS, XPS, Rp, CR, and nanoindentation methods were used for the structural, chemical, electrochemical, and mechanical characterization of the investigated systems.

Our SEM investigation showed large cracks on the surface, especially in samples S3 and S3`, which affect both their corrosion protection ability and their mechanical properties. The comparison of the changes in the concentrations of the elements Ce, Ca, and P, characteristic of the formed conversion layers, registered during EDS analyses, shows that the concentration of Ce changes insignificantly in all studied samples, varying in the range of 2.0–3.1 wt.%. The change in Ca content is about four times greater in samples S3 and S3`, while the change in P concentration is even more pronounced in samples S3 and S3` and to a lesser extent in samples S4 and S4`. As a result of XPS studies, it was found that the concentration of Ce on the surface of S2 is 15.8 at.%, while on the anodized Al—S2`—it is more than fifteen times lower (0.9 at.%). In samples S3 and S3`, the concentration of Ce decreases to 0.5 and 0.4 at.%, respectively, while the concentration of Ca in both samples (non-anodized and anodized Al substrates) is 1.7 at.%. The P concentrations are 11.6 (on a non-anodized substrate) and 17.9 at.% (on anodized substrate). The most probable phases that were formed during the directed preparation of the modeled corrosion protection systems on Al 1050 are as follows: Al_2_O_3_, Се_2_O_3_, and СеО_2_ for S2 and S2`; Al_2_O_3_, AlPO_4_, AlOOH, Се_2_O_3_, CePO_4_, NaH_2_PO_4_, Ca(PO_4_)_3_(OH), and Ca(NO_3_)_2_ for S3 and S3`; AlPO_4_, CePO_4_, PO^3−^, P_2_O_5_(P_4_O_10_), and NaH_2_PO_4_ for S4 and S4`.

The study of the corrosion resistance Rp and the corrosion rate CR of samples S1–S4 shows that sample S1 is characterized by the highest value for Rp at the initial moment of exposure, while the most resistant and reaching the highest value after 168 h of exposure in CM is sample S4. In samples S1–S4`, the highest and relatively weakly changing values for Rp are sample S3`, followed by sample S4’. The study of the mechanical properties of samples S1–S4 shows that their indentation hardness remains almost constant, while for samples deposited on an anodized aluminum substrate (S1`–S4`), the indentation hardness and modulus were much higher than those deposited on a non-anodized aluminum substrate. We found that the thin ceria and phosphate conversion coatings deposited on anodized aluminum Al 1050, which we investigated, not only protect better than uncoated Al 1050 from corrosion but also have improved mechanical properties, which is very important for many applications, such as in automotive catalytic converters, marine applications, and biomedical applications, including total joint replacement, tooth root implantation, and others.

The limitation of the study is that one type of mechanical test (nanoindentation) was performed only on the surface of the samples, but not within the volume. Therefore, in future research, we plan to include additional mechanical testing, such as tensile tests, three-point bending tests, or scratch tests, to complement the hardness evaluations.

## Figures and Tables

**Table 1:** Type of treatment for the samples.

Abbreviation of the Samples	Type of Treatment of the Samples			
	In 15% H_2_SO_4_, 15 °C; i_an_ = 1.5 A.dm^−2^, 48 min	In 0.5 M CeCl_3_ × 7H_2_O + 1 × 10^−5^ M CuCl_2_ × 2H_2_O (25 °C; 2 h)	In 0.5 M NaH_2_PO_4_ + 0.1 M Ca(NO_3_)_2_ Solution (85 °C) 3 × 5 min + 72 h in Humidity Atmosphere	In 0.22 M NH_4_H_2_PO_4_ Solution (85 °C, 5 min)
S1	NO	NO	NO	NO
S1`	YES	NO	NO	NO
S2	NO	YES	NO	NO
S2`	YES	YES	NO	NO
S3	NO	YES	YES	NO
S3`	YES	YES	YES	NO
S4	NO	YES	NO	YES
S4`	YES	YES	NO	YES

**Table 2:** Results of EDS analysis of the changes in element concentrations of the studied systems.

Sample	Al, wt.%	O, wt.%	Fe, wt.%	Ce, wt.%	S, wt.%	Ca, wt.%	Cl, wt.%	P, wt.%	Na, wt.%
S1	97.8 ± 0.58	2.2 ± 8.10^−3^	0	0	0	0	-	-	-
S1`	49.8 ± 0.43	43.2 ± 0.40	0	0	7.0 ± 3.10^−2^	0	-	-	-
S2	86.1 ± 0.52	10.4 ± 0.06	0.4 ± 8.10^−4^	3.1 ± 9.10^−3^	0	-	0	-	-
S2`	37.8 ± 0.17	51.8 ± 0.28	0	2.5 ± 8.10^−3^	3.1 ± 5.10^−3^	-	4.8 ± 9.10^−3^	-	0
S3	68.9 ± 0.41	22.6 ± 0.11	0.4 ± 4.10^−4^	2.2 ± 7.10^−3^	0	0.8 ± 8.10^−4^	0	5.1 ± 1.10^−2^	0
S3`	20.0 ± 0.06	50.3 ± 0.25	0	2.2 ± 6.10^−3^	0	3.1 ± 4.10^−3^	0	21.6 ± 7.10^−2^	2.8 ± 6.10^−3^
S4	87.9 ± 0.56	9.3 ± 0.05	0	2.0 ± 5.10^−3^	0	0	0	0.8	0
S4`	42.5 ± 0.13	46.5 ± 0.16	0	2.3 ± 5.10^−3^	3.9 ± 5.10^−3^	0	2.5 ± 2.10^−3^	2.3 ± 2.10^−3^	0

**Table 3:** Results of XPS analysis of the changes in element concentrations of the studied systems.

Sample	Al, at.%	O, at.%	Ce, at.%	Ca, at.%	P, at.%	Cu, at.%	Na, at.%	N, at.%
S1	39.0 ± 0.4	61.0 ± 0.4	-	-	-	0	-	-
S1`	36.8 ± 0.3	63.2 ± 0.4	-	-	-	0	-	-
S2	11.0 ± 0.1	70.5 ± 0.6	15.8 ± 6.10^−2^	-	-	2.7 ± 4.10^−3^	-	-
S2`	32.0 ± 0.3	66.7 ± 0.6	0.9 ± 3.10^−3^	-	-	0.4 ± 6.10^−4^	0	0
S3	19.6 ± 0.2	65.1 ± 0.4	0.5 ± 2.10^−3^	1.7 ± 3.10^−3^	11.6 ± 0.1	0	0.6 ± 4.10^−4^	1.0 ± 1.10^−3^
S3`	13.1 ± 5.10^−2^	64.4 ± 0.4	0.4 ± 7.10^−4^	1.7 ± 3.10^−3^	17.9 ± 7.10^−2^	0.2 ± 9.10^−5^	0.2 ± 2.10^−4^	2.1 ± 4.10^−3^
S4	7.6 ± 1.10^−2^	76.3 ± 0.7	1.4 ± 4.10^−3^	-	14.1 ± 5.10^−2^	0	0	0
S4`	12.6 ± 3.10^−2^	75.1 ± 0.7	1.3 ± 4.10^−3^	-	6.5 ± 2.10^−2^	0	0	4.5 ± 9.10^−3^

**Table 4:** The most probable phases on the aluminum surface according to the analysis of the studied samples.

Sample	Al	Ce	P	Ca
S2 and S2`	Al_2_O_3_	Ce_2_O_3_+ CeO_2_		
S3 and S3`	Al_2_O_3_; AlPO_4_ AlOOH	Ce_2_O_3_ CePO_4_	NaH_2_PO_4_	Ca_5_(PO_4_)_3_(OH) Ca(NO_3_)_2_
S4 and S4`	AlPO_4_	CePO_4_	PO_3_^−^ P_2_O_5_ (P_4_O_10_) NaH_2_PO_4_	

## Data Availability

The original contributions presented in this study are included in the article. Further inquiries can be directed to the corresponding author.
